# A Novel Case of Severe Respiratory Symptoms and Persistent Pulmonary Hypertension in a Saudi Neonate With SARS-CoV-2 Infection

**DOI:** 10.7759/cureus.10472

**Published:** 2020-09-15

**Authors:** Kumail B Algadeeb, Haider H AlMousa, Sajjad M AlKadhem, Mohammed O Alduhilan, Yameen Almatawah

**Affiliations:** 1 Neonatology, Maternity and Children Hospital, Al-Ahsa, SAU; 2 Pediatrics, Maternity and Children Hospital, Al-Ahsa, SAU; 3 Pediatric Infectious Disease, Maternity and Children Hospital, Al-Ahsa, SAU

**Keywords:** pregnancy, covid-19, sars-cov-2, neonate, case report, saudi arabia, pphn

## Abstract

Coronavirus disease 2019 (COVID-19) is an emergent disease that has spread rapidly to infect more than 210 countries across the world. With the increasing number of infected pregnant women, many physicians hypothesized the perinatal transmission as a potential route of transmission. Some cases of perinatal transmission have been described, but it is unclear if these occurred via the transplacental or the transcervical routes or through environmental exposure. In this report, we described a case of a female infant who was delivered by caesarean section at 34 weeks’ gestation to an infected mother. The neonate was transferred into the Neonatal Intensive Care Unit (NICU) Level 3, with the precaution of airborne and contact isolation. All required investigations were performed, including blood gases, nasopharyngeal swab, chest x-ray, and echocardiogram. On the fifth day of delivery, her investigations demonstrated a positive severe acute respiratory syndrome coronavirus 2 (SARS-CoV-2) infection. Despite applying all recommended guidelines and following the treatment protocol, she developed severe respiratory symptoms with persistent pulmonary hypertension, which progressed significantly to her death.

## Introduction

Coronavirus disease 2019 (COVID-19) has spread rapidly across the world since December 2019, when it initially emerged in Wuhan (Hubei, China), and millions of confirmed cases have been reported worldwide [1]. It has also been named severe acute respiratory syndrome coronavirus 2 (SARS-CoV-2). The number of pregnant women infected with the SARS-CoV-2 virus is increasing. SARS-COV-2 is mainly transmitted through droplets, but other routes have been hypothesized. Some cases of perinatal transmission have been described, but it is unclear if these occurred via the transplacental or the transcervical routes or through environmental exposure [2-4]. As far as we know, there have been only two reports with effective investigation proving the vertical transmission in neonates born to mothers with positive perinatal SARS-CoV-2 [5,6].

Since the emergence of the pandemic in March 2020, 45 pregnant women with positive reverse transcription polymerase chain reaction (RT-PCR) results for SARS-CoV-2 have been admitted into our institute, Maternity and Children’s Hospital in Al-Ahsa, Saudi Arabia. All of their newborns were screened for SARS-CoV-2 following the Ministry of Health COVID-19 protocol [7]. There were only three neonates with positive results for the test by nasopharyngeal RT-PCR. Notably, only one of these three neonates was symptomatic and required advanced intensive care management, and this is the case that we are reporting in this paper.

## Case presentation

A 37-year-old primigravida woman visited our obstetric emergency department on June 2020, at 31 weeks’ gestation with intermittent bloody spotting and leg swelling. She had a history of pregnancy-induced hypertension on methyldopa treatment and gestational diabetes controlled through her diet. Fundal ultrasonography showed a low-lying placenta. The patient was admitted for bleeding observation and medical management. She completed a dexamethasone course six days after admission, and the course was not repeated.

**Figure 1:**
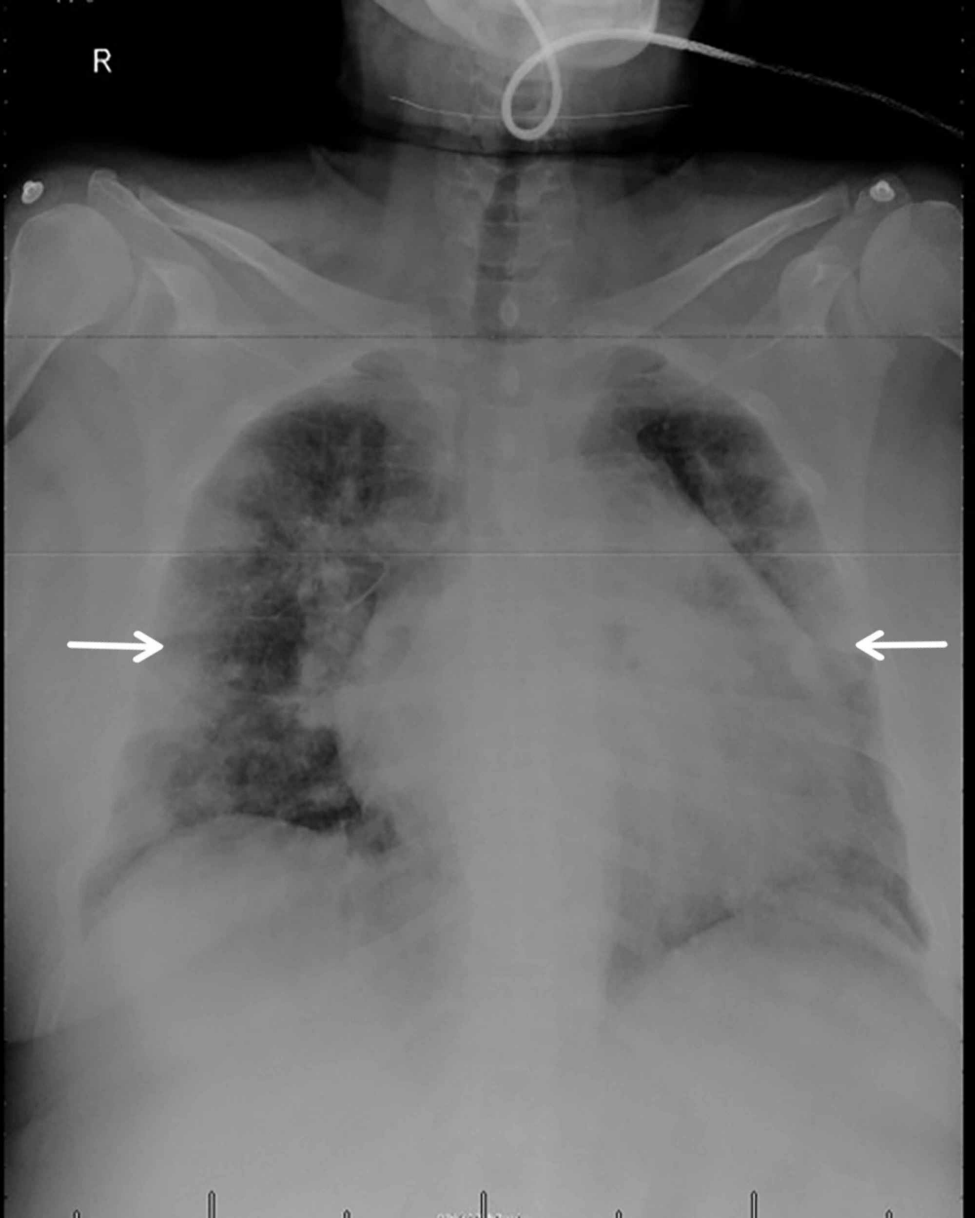
Chest x-ray of the mother with positive SARS-CoV-2 infection shows bilateral multifocal peripheral airspace opacities. SARS-CoV-2, severe acute respiratory syndrome coronavirus 2

One day before the delivery, the patient experienced shortness of breath and fever (38˚C). On examination, she demonstrated harsh bronchial breathing on chest auscultation, and she required low-flow oxygen to maintain her oxygen saturation above 90%. Her chest x-ray showed bilateral multifocal peripheral airspace opacities, suggesting COVID-19 (Figure 1).

She was isolated immediately, and a nasopharyngeal swab for SARS-CoV-2 PCR testing was taken. A high vaginal swab and urine culture were negative for bacterial growth, and there were no signs of chorioamnionitis. Cardiotocography (CTG) was not reassuring, with a decrease in beat-to-beat variability; therefore, an emergency caesarean was planned.

On the day of delivery, a female infant was delivered by caesarean section at 34 weeks’ gestation, with a birth weight of 2,475 g (on the 70th percentile), head circumference of 30 cm (on the 22nd percentile), and length of 43 cm (on the 25th percentile). She cried immediately after birth, and the Apgar score was 8 and 8 at 1 and 5 minutes, respectively. The baby was intubated at 15 minutes of age, and positive pressure ventilation was provided because she was grunting, severely retracting, and having low oxygen saturation despite application of continuous positive airway pressure (CPAP) and a fraction of inspired oxygen (FiO_2_) of 100%. The baby shifted to the Neonatal Intensive Care Unit (NICU) Level 3, with the precaution of airborne and contact isolation. All staff who attended the delivery were following full precautions as per the protocol for suspected or confirmed COVID-19 cases.

**Figure 2:**
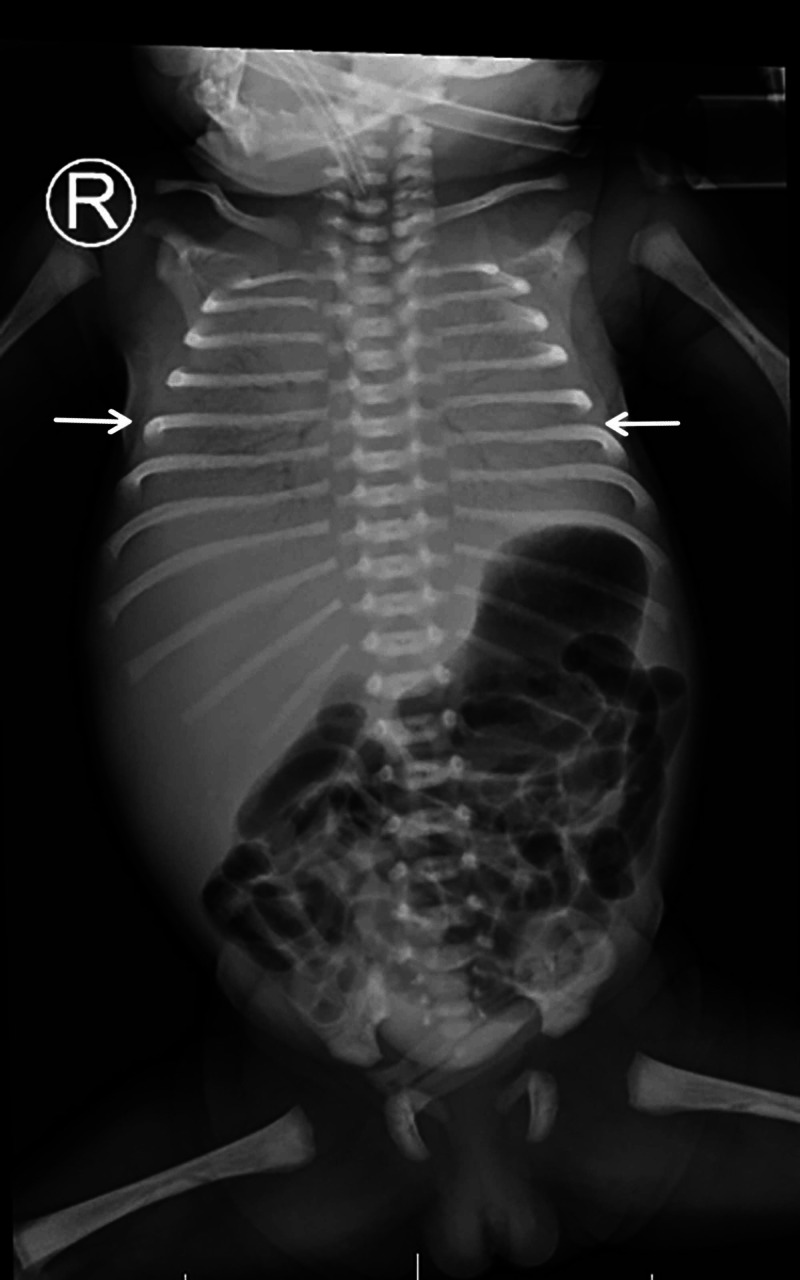
First chest x-ray of the neonate shows bilateral ground-glass appearance suggesting respiratory distress syndrome. An endotracheal tube was high in this x-ray.

In the NICU, the neonate was sedated and mechanically ventilated, requiring high ventilatory pressures and FiO_2_ of 100%, with a differential saturation of more than 10 between pre- and post-ductal oxygen saturations. Even with higher settings, it was difficult to ventilate and oxygenate the baby optimally, and hence baby was shifted to high-frequency ventilation with a mean airway pressure of 17 cm H_2_O, a frequency of 15 Hz, and an amplitude of 30 cm H_2_O. The first x-ray of the chest showed the ground-glass appearance of respiratory distress syndrome (Figure 2).

**Figure 3:**
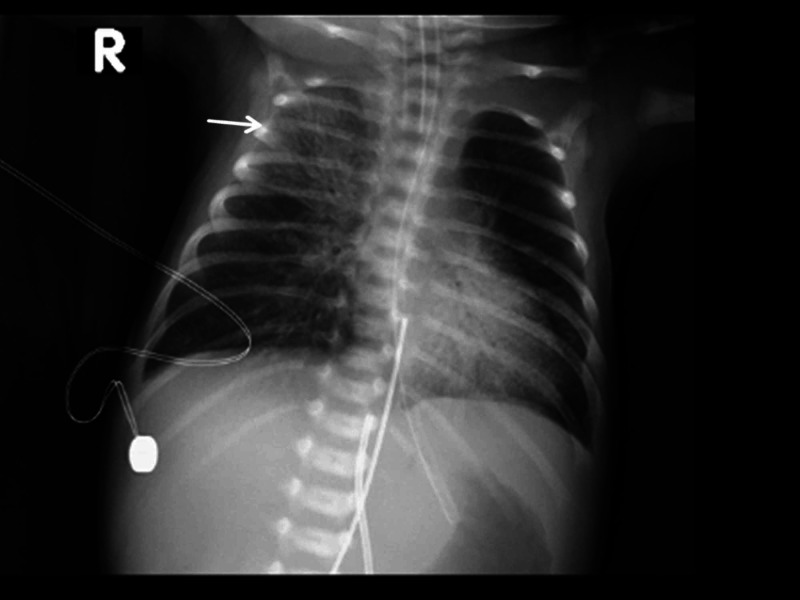
Chest x-ray of the neonate at day 2 of life shows bilateral perihilar hazy opacities with bronchial wall thickness, more evident at the left side with mildly hyperinflated lungs.

The baby received two doses of surfactant through the endotracheal tube, but there was no improvement in the ventilator setting or FiO_2_ requirement. Repeated chest x-rays showed bilateral perihilar hazy opacities with bronchial wall thickness, more evident on the left side, with mildly hyperinflated lungs (Figure 3).

The neonate’s blood gas showed an element of metabolic acidosis, with a PO_2_ of 77 and an oxygen index of 23. The electrocardiogram was normal, and no differences were noticed in four limb blood pressure readings. The echocardiogram indicated severe persistent pulmonary hypertension, severe tricuspid regurgitation, and moderate mitral regurgitation with an estimated pulmonary pressure of 76 mmHg, a bidirectional shunt seen at the level of the patent foramen ovale, and a right-to-left shunt through the large patent ductus arteriosus. There was a normal ventricular function with an ejection fraction of 66% and fractional shortening of 34%. Therefore, the neonate started on inhaled nitric oxide at a dose of 20 ppm, and by the end of the first 24 hours of life, her circulation was being supported with 15 µg/kg/min of dobutamine and 15 µg/kg/min of dopamine to maintain her mean blood pressure above 40 mmHg.

The baby remained nil by mouth and started on full total parenteral nutrition with blood sugar and frequent laboratory monitoring (Table 1). The infectious disease team was involved, and sepsis workup was performed. The neonate was started on ampicillin and cefotaxime. Cultures of blood and cerebrospinal fluid showed negative for bacterial growth. C-reactive protein had a positive value of 6 mg/L.

**Table 1:** Basic laboratory result for the neonate. Hgb: haemoglobin, WBC: white blood cell, PTT: partial thromboplastin time, INR: international normalized ratio, ALT: alanine aminotransferase, AST: aspartate transaminase, SrCr: serum creatinine, BUN: blood urea nitrogen.

Laboratory result at first day			Laboratory result at 24 hours prior death		Reference range
Complete blood count	Hgb (g/dL)	15	Hgb (g/dL)	14	12.5-13.7 g/dL
	WBC (×10^3^/µL)	26	WBC (×10^3^/µL)	23	4.5-13.5 ×10^3^/µL
	Platelets (×10^3^/µL)	70	Platelets (×10^3^/µL)	64	150-350 ×10^3^/µL
Coagulation profile	PTT (seconds)	44	PTT (seconds)	43	30-40 seconds
	INR	1	INR	1	0.8-1.1
Electrolytes	Na (mmol/L)	129	Na (mmol/L)	152	135-145 mmol/L
	Ca (mmol/L)	1.9	Ca (mmol/L)	2.2	2.1-2.5 mmol/L
	K (mmol/L)	5	K (mmol/L)	4.8	3.5-5 mmol/L
	PO_4_ (mmol/L)	2	PO_4_ (mmol/L)	1.6	0.78-1.42 mmol/L
Renal profile	SrCr (µmol/L)	98	SrCr (µmol/L)	132	44-88 µmol/L
	BUN (mmol/L)	3	BUN (mmol/L)	11	1.7-7.1 mmol/L
Liver profile	ALT (unit/L)	8.8	ALT (unit/L)	48	10-30 unit/L
	AST (unit/L)	41	AST (unit/L)	23	13-35 unit/L
	Total bilirubin (mg/dL)	5.9	Total bilirubin (mg/dL)	19	0.2-1.5 mg/dL

At the third day of life, the mother was confirmed to be SARS-CoV-2 positive; therefore, nasopharyngeal and tracheal swabs for SARS-CoV-2 were taken from the neonate for PCR testing, which revealed a positive result by the fifth day of life. Our hospital follows the Supportive Care and Treatment Protocol of the Saudi Ministry of Health for Patients Confirmed with COVID-19, which recommends using hydrocortisone for pre-term infants with a corrected gestation age of <40 weeks: (IV) 0.5 mg/kg every 12 hours for seven days and then 0.5 mg/kg once daily for three days [7]. This was the protocol followed in this case.

The creatinine level rose, accompanied by a decrement in the urine output, indicating acute kidney injury. There was a substantial drop in the platelet count during the hospital stay, and the baby required frequent platelet transfusions. She also required a blood transfusion once. The coagulation profile was normal. By day 6, the neonate developed an abnormal cyclic movement in all limbs associated with decreasing oxygen saturation and tachycardia; therefore, she was loaded with phenobarbital followed by maintenance doses. A brain ultrasound showed grade ΙΙΙ germinal matrix haemorrhage with mild dilatation in lateral ventricles.

Over the following days, the neonate remained critically ill with cardiorespiratory failure, persistent pulmonary hypertension, and acute kidney injury. Antibiotics were upgraded to imipenem/cilastatin and vancomycin. SARS-CoV-2 PCR testing was repeated twice 72 hours apart, and the results were positive in all samples. She was on high-frequency ventilation and nitric oxide, with low oxygen saturation and poor perfusion supported by dopamine, dobutamine, and epinephrine. At day 11, she developed bradycardia, and cardiopulmonary resuscitation was initiated; however, she died.

## Discussion

The SARS-CoV-2 novel virus is known to cause severe pneumonia in adults. The early neonatal infection has been reported frequently, but the reports by Vivanti et al. and Patane et al. are probably the first and only reports confirming vertical transmission [5,6]. In our centre, we have confirmed SARS-CoV-2 infection in 45 pregnant women until the date of this report. Three neonates’ PCR test results were positive for the virus: aside from our reported case, another two neonates were asymptomatic. This accounts for a 15% incidence among infected mothers, compared to 9% found by Zhu et al. [3], and no cases reported by Schwartz [8], taking the limited sensitivity of nasopharyngeal RT-PCR testing into consideration [9].

In reviewing the presentation of coronavirus among different settings and ages, the clinical picture varies from asymptomatic carriers, severe pneumonia, shock, and thromboembolism to multisystem inflammatory syndrome in children (MIS-C). Neonates have been reported to be asymptomatic or to have mild respiratory symptoms or mild pneumonia [4]. One case was reported to have meningoencephalitis [6].

To date, there have been no reported cases of a neonate with severe respiratory symptoms and a severe picture of pneumonia as in our case. The case also exhibited severe persistent pulmonary hypertension that responded poorly to inhaled nitric oxide and respiratory support. The severity of vertical infection of SARS-CoV-2 may depend on advancing gestational age, with less infectivity early in pregnancy and a more severe picture, as in our case, associated with late-trimester infection. 

Although MIS-C has been highlighted in children infected with the SARS-CoV-2 virus and has been linked to poor prognosis [10], the multiple organ dysfunctions that the neonate in our case study had can be explained by her severe persistent pulmonary hypertension status and hemodynamic instability, especially with lack of skin manifestation, fever, and other signs and symptoms that exist in MIS-C.

In our reported case, the mother was infected and symptomatic shortly before delivery. A caesarean section was performed due to bleeding and abnormal CTG. Our healthcare workers were strictly following the infection-control guidelines related to COVID-19 and followed the pathway for suspected or confirmed COVID-19 cases, which includes the assignment of a trained team from all related subspecialties to attend the delivery, the limitation of healthcare workers to a minimum in a designated isolated operating room, and the use of an N95 mask by everyone. The operation was held under spinal anaesthesia, and no arousal-generating procedures were needed. The mother was wearing a surgical mask because she was suspected of having SARS-CoV-2 infection. Resuscitation of the neonate was conducted 3 m away from the operation site, with full personal protective equipment (PPE). Delayed cord clamping was not applied, and no skin-to-skin contact was allowed between the mother and the newborn. The transferring team was wearing full PPE, and the patient had been in airborne precaution since admission to NICU Level 3. None of the healthcare providers who dealt with the neonate or her mother had symptoms of COVID-19.

The application of all these measures and precautions suggests that the SARS-CoV-2 infection in the neonate was caused by vertical transmission, although droplet transmission cannot be ruled out. Further investigating tools such as sampling the amniotic fluid for the virus, a placenta pathology examination, and a neonate blood sample test for antigens and antibodies would have been required to validate a vertical transmission claim.

Neonatal pneumonia has been reported in babies born to COVID-19 mothers [3,9]. In the Zhu study [3], 6 out of 10 infants born to COVID-19 mothers were experiencing respiratory symptoms. Moreover, seven babies have x-ray findings. This includes pneumonia in four neonates, bilateral blurred lungs and pneumothorax in one neonate, and ground-glass appearance in two other neonates. Although all their RT-PCR testing came negative for SARS-CoV-2, small sample size and high rate of false-negative were considered. In our reported case, ground-glass appearance and lung infiltrate may indicate a severe form of a similar pathological picture that lead to the unfortunate outcome. Other differential diagnoses, such as late prematurity with the risk of respiratory distress syndrome or transient tachypnea of the newborn, cannot solely explain the scenario, although it may have worsened the condition and resulted in this severe picture of pneumonia and persistent pulmonary hypertension. Interestingly, angiotensin-converting enzyme 2 (ACE2), which has been recorded as a targeted receptor for SARS-COV-2 [11], plays an important role in the pathogenesis of a variety of lung injuries in a neonate. Furthermore, it has been hypothesized that ACE2 has a role in vascular remodelling and pulmonary hypertension [12]. The claim of whether COVID-19 contributed directly to the pathology of pulmonary hypertension or through lung pneumonia needs to be investigated.

## Conclusions

Owing to the quick deterioration of the neonate, SARS-CoV-2 does not appear to be a benign entity in neonates. It could be very well stated that COVID-19 may be considered as a possible cause of severe pneumonia and subsequently the mortality. Although not the usual presentation, it could have been secondary to a severe course of respiratory distress syndrome of prematurity or other comorbidities, such as primary persistent pulmonary hypertension of the newborn.
